# ISL1, a novel regulator of *CCNB1*, *CCNB2* and *c-MYC* genes, promotes gastric cancer cell proliferation and tumor growth

**DOI:** 10.18632/oncotarget.9269

**Published:** 2016-05-10

**Authors:** Qiong Shi, Weiping Wang, Zhuqing Jia, Ping Chen, Kangtao Ma, Chunyan Zhou

**Affiliations:** ^1^ Department of Biochemistry and Molecular Biology, School of Basic Medical Sciences, Beijing Key Laboratory of Protein Posttranslational Modifications and Cell Function, Key Laboratory of Molecular Cardiovascular Sciences, Ministry of Education of China, Peking University, Beijing, P.R. China

**Keywords:** ISL1, gastric cancer, proliferation, CCNB, c-MYC

## Abstract

*I*slet-1 (*ISL1*) belongs to the LIM homeodomain transcription factor family, which is specifically expressed in certain tissue types only. Previously, we reported that *ISL1* is aberrantly overexpressed in gastric cancer (GC). However, its role in GC is not clear. Here, we report that *ISL1* is aberrantly upregulated not only in human gastric carcinoma tissues but also in some GC cell lines. Upregulated *ISL1* expression enhanced xenografted gastric carcinoma development, while *ISL1* knockdown inhibited GC growth in nude mice. *ISL1* overexpression promoted GC cell proliferation, colony formation, and cell growth in soft agar, and facilitated cell cycle transition in GC cells, demonstrated an increase in the proportion of cells in the G_2_/M and S phases and a decrease in the proportion of cells in the G_1_ phase. Furthermore, we provide evidence that ISL1 is a novel regulator of the cyclin B1 (*CCNB1*), cyclin B2 (*CCNB2*) and c-myc (*c-MYC*) genes. ISL1 activated the expression of these genes in GC cells by binding to the conserved binding sites on their promoters or enhancers. The expression levels of the genes were decreased in response to ISL1 knockdown. Therefore, ISL1 may serve as a potential therapeutic target in GC.

## INTRODUCTION

Gastric cancer (GC) is the fifth most common cancer and the third leading cause of cancer death worldwide [1]. Like most malignant tumors, GC malignancy is caused by uncontrolled cell proliferation, invasion and metastasis [2]. Although there have been many advances in GC treatment, the molecular mechanisms that govern GC malignancy and progress remain to be explored.

Islet-1 (ISL1), a LIM-homeodomain transcription factor, was initially cloned from rat pancreatic insulin-producing cells, where it binds the insulin gene enhancer [3]. ISL1 plays a key role in the development of specialized cells in multiple tissue types, including nervous system, skeletal muscle, heart, kidneys, and endocrine organs such as the pituitary gland and pancreas [4]. Previous studies have demonstrated that ISL1 is a marker for well-differentiated pancreatic neuroendocrine tumors [5]. Recent studies have identified ISL1 as a sensitive lineage-specific marker for pancreatic neuroendocrine neoplasms and their metastases [6], and it is commonly expressed in rhabdomyosarcoma [7]. We recently reported that *ISL1* is highly expressed in GC and is correlated with advanced tumor-nodes-metastasis stage, lymph node metastasis, and poorer overall survival [8]. However, the role and detailed mechanism of ISL1 in cancer development remain unknown.

In the present study, we investigated whether ISL1 plays an oncogenic role in human GC. We demonstrated that ISL1 enhanced tumor growth *in vivo* and promoted GC cell proliferation, colony formation, and soft agar growth *in vitro*. We found that ISL1 targeted three downstream genes: cyclin B1 (*CCNB1*), cyclin B2 (*CCNB2*) and c-myc (*c-MYC*), in GC cells. *ISL1* knockdown resulted in cell cycle delay, and mutation of the ISL1 binding site on the putative target genes impaired the transcriptional activation mediated by ISL1. Our data suggest that aberrantly expressed *ISL1* may stimulate *CCNB1*, *CCNB2* and *c-MYC* expression and therefore play an important role in gastric carcinogenesis.

## RESULTS

### ISL1 was highly expressed in GC cells

**Figure 1:**
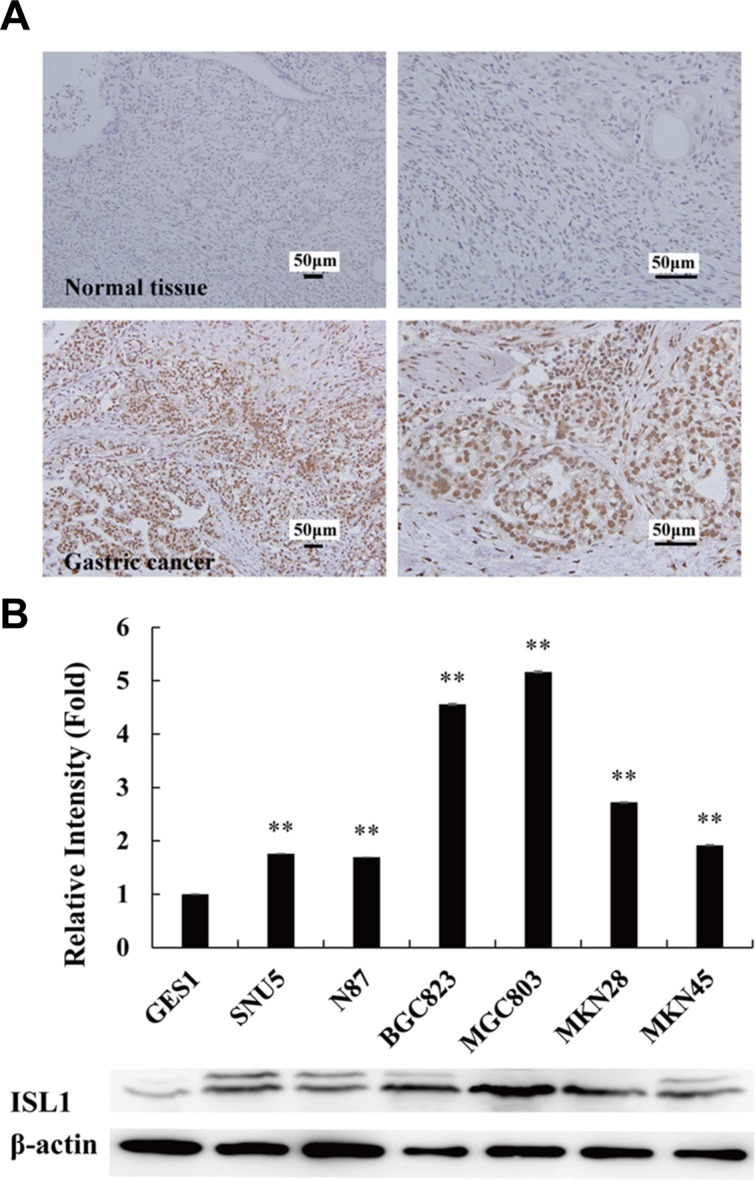
Aberrant expression of ISL1 in GC tissues **(A)** Representative IHC stainings of ISL1 expression in normal gastric mucosa (top, *n* = 12) and GC samples (bottom, *n* = 24). **(B)** Grayscale scanning analysis results of ISL1 expression in the tested cell lines. Grayscale scanning was performed on the western blots of three independent experiments for quantitative analysis. Representative western blots of three experiments are shown at the bottom. β-Actin served as the internal control. GES1, normal gastric cell line; others, GC cell lines. Bars represent the means ± SD (**p < 0.01 vs. GES1 cells).

We previously reported the aberrant expression of ISL1 in GC tissues [8]. In the present study, we demonstrate that *ISL1* expression was upregulated in 24 GC biopsies (18 poorly differentiated adenocarcinoma, 4 moderately differentiated adenocarcinoma, 2 well-differentiated adenocarcinoma) by immunohistochemistry (IHC) (12 normal gastric tissues were used as the control). Representative images of IHC staining are shown in Figure 1A. We also examined ISL1 expression by western blot analysis in six GC cell lines; a normal human gastric epithelium cell line was used as the control. Grayscale scanning of the western blots of three independent experiments revealed that ISL1 expression was highly upregulated in the GC cell lines (Figure 1B), and its level was negatively correlated with the cell differentiation grades, i.e., ISL1 levels were lower in higher–differentiation grade cells. It should be mentioned that ISL1 was visualized as two bands in the western blots of some samples. ISL1 has an alternatively spliced variant [9]. These two bands may represent the alternatively spliced variants, ISL1 α and ISL1 β.

### ISL1 promoted colony formation, soft agar growth and tumor growth

**Figure 2:**
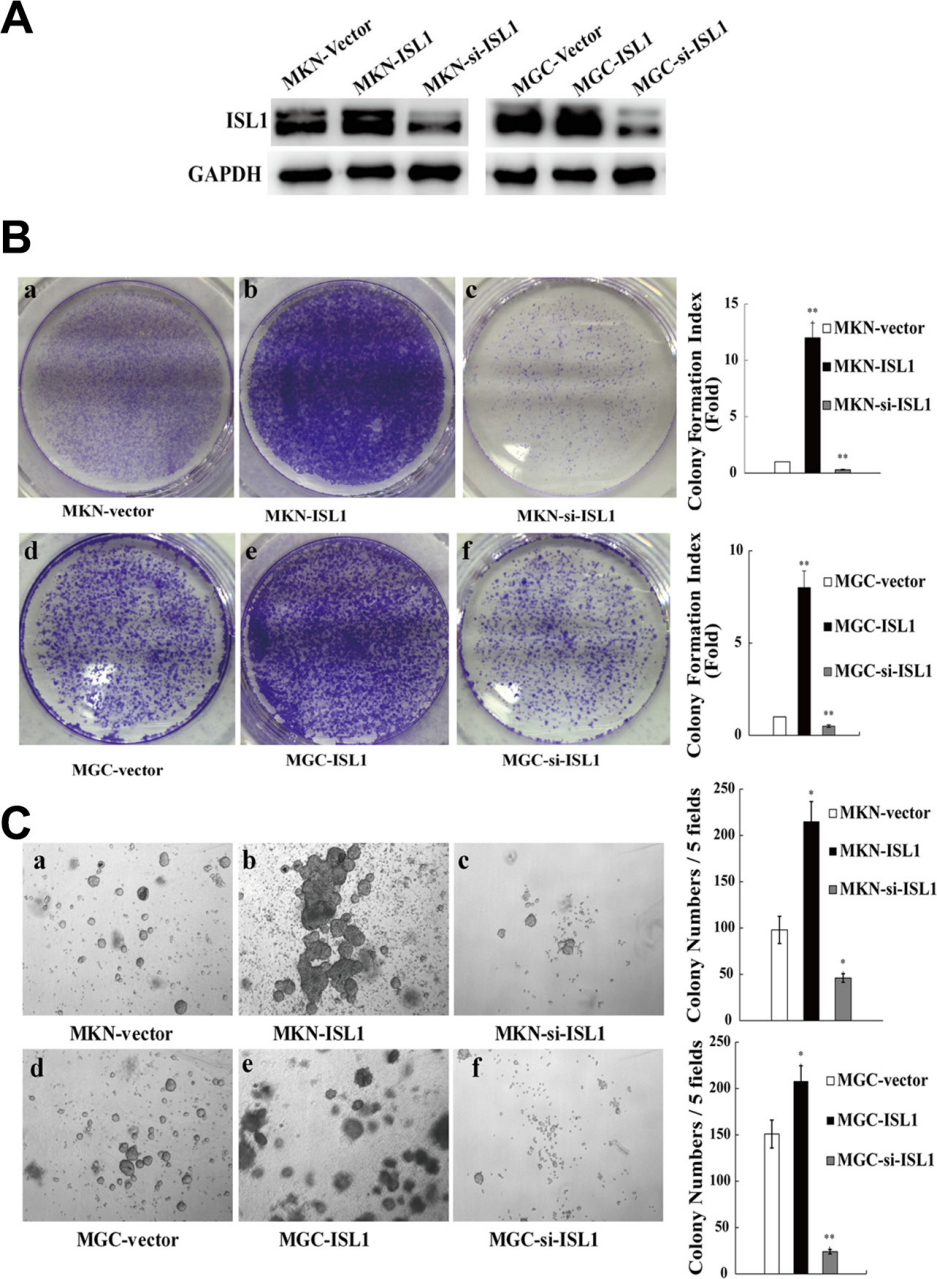
ISL1 promoted colony formation *in vitro* **(A)** Western blotting analysis of ISL1 levels in MKN28 and MGC803 cell lines with stable ISL1 overexpression (ISL1) or knockdown (si-ISL1) by transfection with pcDNA3.1-ISL1 and pLL3.7-ISL1-siRNA plasmids (cells transfected with pcDNA3.1 vector were used as the negative control), respectively. GAPDH levels served as the internal control. **(B)** PCF assay performed to determine the proliferation of MKN28 or MGC803 cells stably transfected with ISL1, si-ISL1, or the vector control (a–f). The quantification results are presented on the right. **(C)** SACF assay of MKN28 and MGC803 cells stably transfected with ISL1, si-ISL1, or vector (a–f). The quantification results are presented on the right. The data are the means ± SD from three independent experiments, each performed in triplicate. (**p < 0.01, *p < 0.05 vs. control).

Previously, we proved that ISL1 promoted the proliferation of adult pancreatic islet cells and lymphoma cells [10, 11]. To determine the role of ISL1 in GC, we established stable ISL1-overexpressing and knockdown MKN28 and MGC803 (thereafter labeled as MGC) cell lines using pcDNA3.1-ISL1 and pLL3.7-ISL1-siRNA plasmids, respectively (Figure 2A). The colony formation assay revealed a significantly increased colony formation index of ISL1-overexpressing cells (MGC–ISL1 cells, 8.0 ± 0.91-fold; MKN28–ISL1 cells, 12.1 ± 1.32-fold) as compared with the vector-transfected control cells (*p* < 0.01, Figure 2B). The soft agar growth assay also revealed significantly increased colony numbers by the ISL1-overexpressing cells (MGC–ISL1, 208 ± 25.1; MKN28–ISL1, 215 ± 18.7) as compared with the vector-transfected control cells (MGC–vector, 151 ± 17.2; MKN28–vector, 98 ± 10.5) (*p* < 0.05, Figure 2C). Conversely, decreased colony formation index and colony number were observed in ISL1-knockdown cells (colony formation index: MGC–si-ISL1, 0.5 ± 0.07-fold; MKN28–si-ISL1, 0.3 ± 0.05-fold, *p* < 0.01; colony number: MGC-si-ISL1, 24 ± 3.5, *p* < 0.01; for MKN28-si-ISL1, 46 ± 5.9, *p* < 0.05) as compared with the control cells (Figure 2B, 2C).

**Figure 3:**
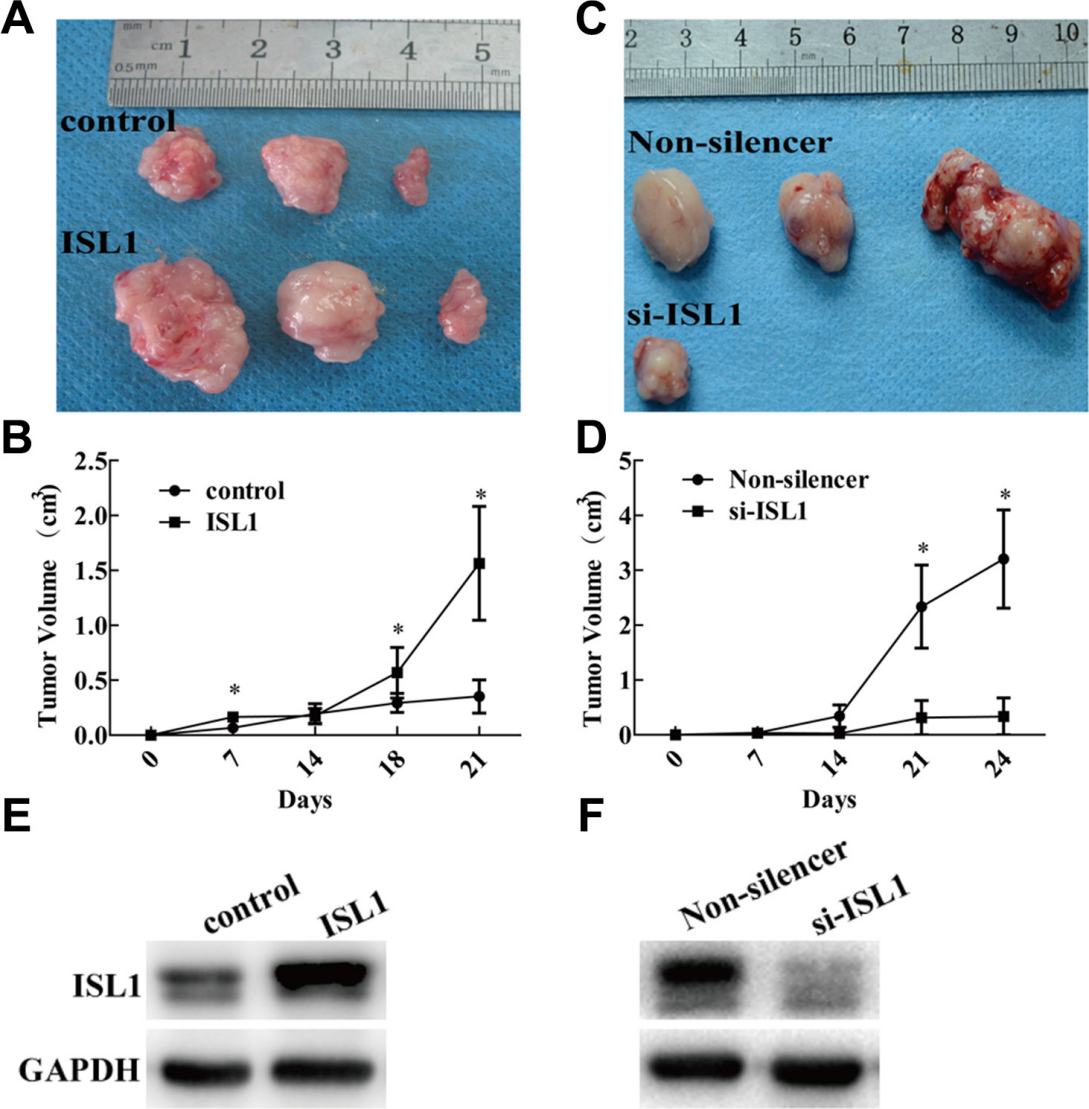
ISL1 promoted tumor growth in nude mice (**A, B**) MGC803 cells stably transfected with pcDNA3.1 (control), pcDNA3.1-ISL1 (ISL1), (**C, D)** MGC803 cells stably transfected with pLL3.7-Non-silencer (Non-silencer), or pLL3.7-ISL1-siRNA (si-ISL1). A total 1 × 10^7^ appropriate cells suspended in 200 μl PBS were injected subcutaneously into the right oxter flank of BALB/c nude mice. Tumors isolated at day 21 (ISL1 and control) or day 24 (si-ISL1 and Non-silencer) are shown in A and C Tumor size was measured at the indicated days post-injection and the results are shown in B and D (*n* = 3 at every time point in each group, *p < 0.05 vs. control or Non-silencer). (**E, F)**Western blot detection of ISL1 levels in tumor samples. GAPDH levels served as the internal control.

To investigate whether ISL1 promotes tumor growth *in vivo*, we used a nude mouse xenograft model to study the impact of ISL1 on GC development. A total 1 × 10^7^ MGC803 cells (stable ISL1-overexpressing or ISL1-knockdown cells and their respective controls, Supplementary Figure S1) were subcutaneously injected into the mice to establish a solid tumor model. Tumor size was measured on the indicated days after injection, and the tumor volumes formed by the ISL1-overexpressing cells were significantly larger than that of the control cells at day 21 (1.56 ± 0.73 cm^3^ vs. 0.35 ± 0.21 cm^3^, *p* < 0.05) (Figure 3A, 3B). Meanwhile, the tumor volumes formed by the ISL1-knockdown cells were obviously reduced at day 24 (0.34 ± 0.48 cm^3^ vs. 3.20 ± 1.26 cm^3^, *p* < 0.05) (Figure 3C, 3D). Next, we compared *ISL1* expression in the tumors isolated from each group at day 21 (ISL1 and control groups) or day 24 (si-ISL1 and Non-silencer groups) by western blotting. ISL1 protein expression levels in the tumors were positively correlated with tumorigenesis in each group (Figure 3E, 3F). These results indicate that ISL1 potentiates GC growth *in vivo*.

### ISL1 promoted cell proliferation

**Figure 4:**
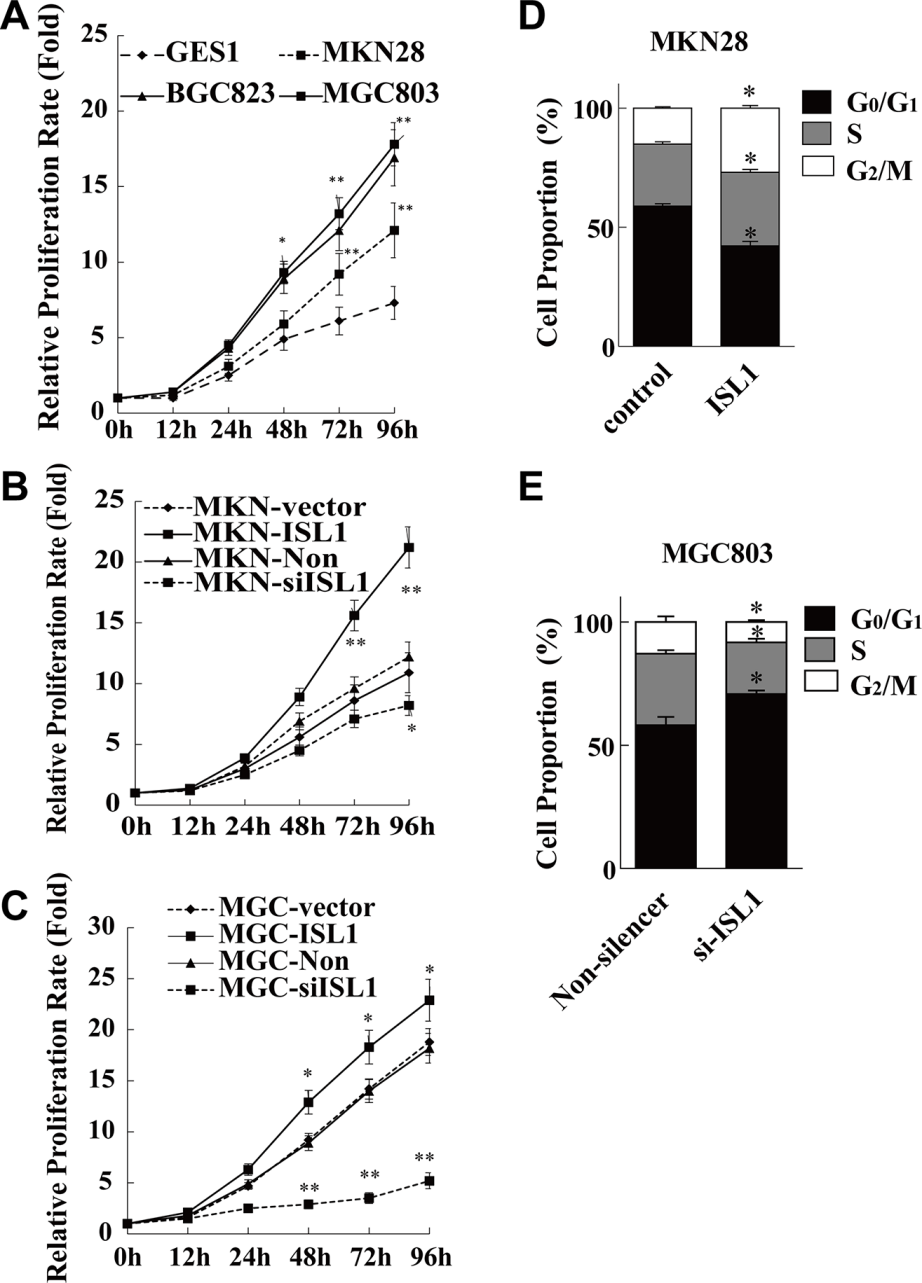
ISL1 promoted GC cell proliferation (**A–C)** The cell proliferation rate as determined using CCK-8 assay. The values at each time point are normalized to that of the relative cells at time 0, which was set as 1. The data are the means ± SD from three independent experiments, each performed in triplicate (**p < 0.01, *p < 0.05 vs. control or Non-silencer at each time point). (**D, E)** Flow cytometry analysis of MKN28 and MGC803 cell cycle distributions. Values are the means ± SD of three independent experiments, each performed in triplicate (*p < 0.05 vs. control or Non-silencer). Control, ISL1, Non-silencer and si-ISL1 represent cells transfected with pcDNA3.1, pcDNA3.1-ISL1, pLL3.7-Non-silencer and pLL3.7-ISL1-siRNA plasmids, respectively.

To further confirm the role of ISL1 in GC cells, we detected proliferation rate of the GES1, MKN28, BGC823, and MGC803 cells, with the levels of *ISL1* expression from low to high as showed in Figure 1B and 1C. Cells were plated in 96-well plates at an initial density of 1000 cells/well. The growth curve was drawn according to the Cell Counting Kit-8 (CCK-8) assay results. The cell proliferation rate was positively correlated with *ISL1* expression levels, i.e., cell proliferation was faster in the three GC cell lines (17.8 ± 1.16 fold for MGC803, 16.9 ± 1.92 fold for BGC823, 12.1 ± 1.12 fold for MKN28) as compared with the normal GES1 cells (7.3 ± 0.58-fold) after 96-h culture (*p* < 0.05 for MKN28, *p* < 0.01 for BGC823 and MGC803, Figure 4A). Consistently, *ISL1* overexpression in MKN28 and MGC803 cells further promoted cell proliferation rates at 96 h after transfection, and *ISL1* knockdown significantly reduced cell proliferation rates (*p* < 0.05, Figure 4B, 4C). The cell cycle profiles of the MKN28 cells demonstrated that *ISL1* overexpression decreased the cell population in the G_1_ phase (from 58.82% ± 1.50 to 42.18% ± 2.67, *p* < 0.05) and increased the cell population in the S and G_2_/M phases (from 26.04% ± 1.29 to 30.81% ± 1.77, and from 15.13% ± 0.69 to 27.00% ± 1.46, respectively, *p* < 0.05). Conversely, *ISL1* knockdown increased the proportion of MGC803 cells in the G_1_ phase (from 58.12% ± 4.76 to 70.71% ± 2.17, *p* < 0.05) and a decreased proportion of cells in the S and G_2_/M phases (from 29.06% ± 1.83 to 21.03% ± 2.08, and from 12.81% ± 3.18 to 7.68% ± 0.77, respectively, *p* < 0.05) (Figure 4D, 4E). These results indicate that ISL1 facilitates the cell cycle and thus promotes GC cell proliferation.

### ISL1 bound to *CCNB1*, *CCNB2* and *c-MYC*

**Figure 5:**
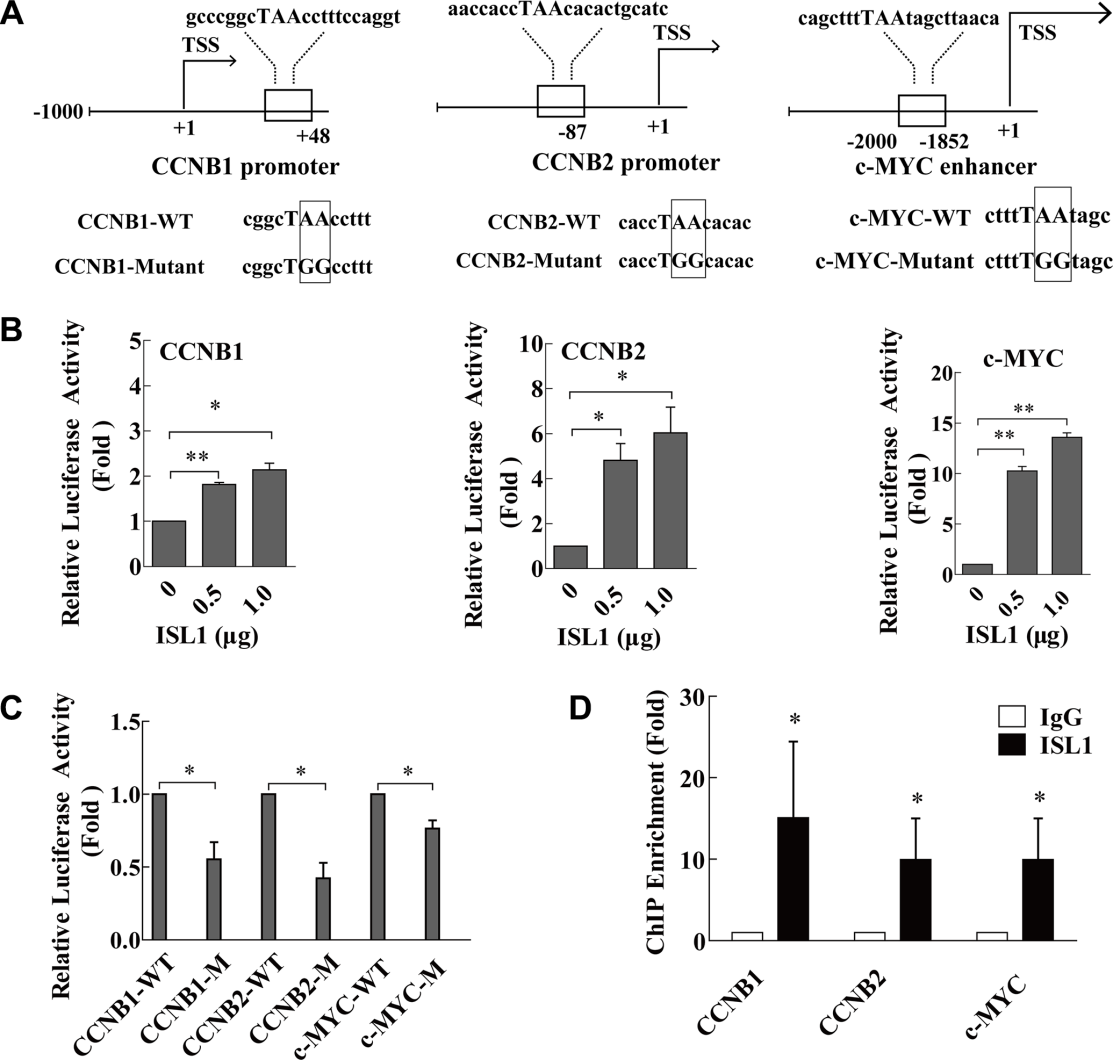
ISL1 bound to *CCNB1*, *CCNB2* and *c-MYC* **(A)** MatInspector software analysis of the consensus binding site (TAAT box) for ISL1 on the human *CCNB1* promoter, the *CCNB2* promoter, and the *c-MYC* enhancer. Sequences containing mutant base pairs (boxes) were used to construct the luciferase reporter constructs mutant-CCNB1-luc, mutant-CCNB2-luc, and mutant-c-MYC-luc. (**B, C**) Luciferase reporter assay of ISL1 transcriptional activity on the luciferase reporter constructs (-luc) of *CCNB1*, *CCNB2* and *c-MYC* in MKN28 cells (WT, wild-type plasmid; M, mutant plasmid). Luciferase activity on the reporter constructs was normalized to *Renilla* luciferase activity. **(D)** ChIP assay was performed with anti-ISL1 antibody using chromatin harvested from MKN28 cells. Extracted DNA was amplified using primers that cover the ISL1 binding sites on the *CCNB1* and the *CCNB2* promoters and the *c-MYC* enhancer by real-time PCR; normal IgG was used as the control. Data represent three independent experiments, each performed in triplicate. Bars represent the means ± SD (*p < 0.05, **p < 0.01 vs. control).

ISL1 is a transcription factor that binds directly to the regulatory sequences of its downstream genes to activate their expression, and the study has reported that ISL1 bound preferentially to the consensus sequence TAAT [12]. To investigate whether ISL1 activates transcription, we analyzed candidate genes selected from the TRANSFAC^®^ 7.0 database (http://www.gene-regulation.com/pub/databases.html), and performed TAAT box consensus binding site analysis on the promoters or enhancers of these genes. Based on these analyses, *CCNB1*, *CCNB2* and *c-MYC* were identified as the likeliest putative direct targets of ISL1. The conserved ISL1 binding site (TAAT) were found at +46 to +48 bp downstream of the ATG translation start site on the *CCNB1* promoter region, −89 to −87 bp upstream of the ATG translation start site on the *CCNB2* promoter region, and at −1856 to −1852 bp upstream of the ATG translation start site on the *c-MYC* enhancer region (Figure 5A). We constructed luciferase reporters for the *CCNB1* promoter (CCNB1-luc), the *CCNB2* promoter (CCNB2-luc), and the *c-MYC* enhancer (c-MYC-luc), which contained the ISL1 binding site on the promoter or enhancer sequences. Following transfection into MKN28 cells, the stimulated activity of CCNB1-luc, CCNB2-luc, and c-MYC-luc was dose-dependent (Figure 5B). Additionally, we established constructs containing mutant ISL1 binding site sequences, designated mutant-CCNB1-luc, mutant-CCNB2-luc, and mutant-c-MYC-luc (Figure 5A). These mutants exhibited significantly decreased luciferase activity compared to the wild-type reporters, indicating that ISL1 plays a key role in activating these genes (Figure 5C). Furthermore, the chromatin immunoprecipitation (ChIP) results confirmed ISL1 binding to the *CCNB1* promoter, the *CCNB2* promoter and the *c-MYC* enhancer (Figure 5D).

### ISL1 upregulated endogenous expression of *CCNB1, CCNB2* and *c-MYC*

**Figure 6:**
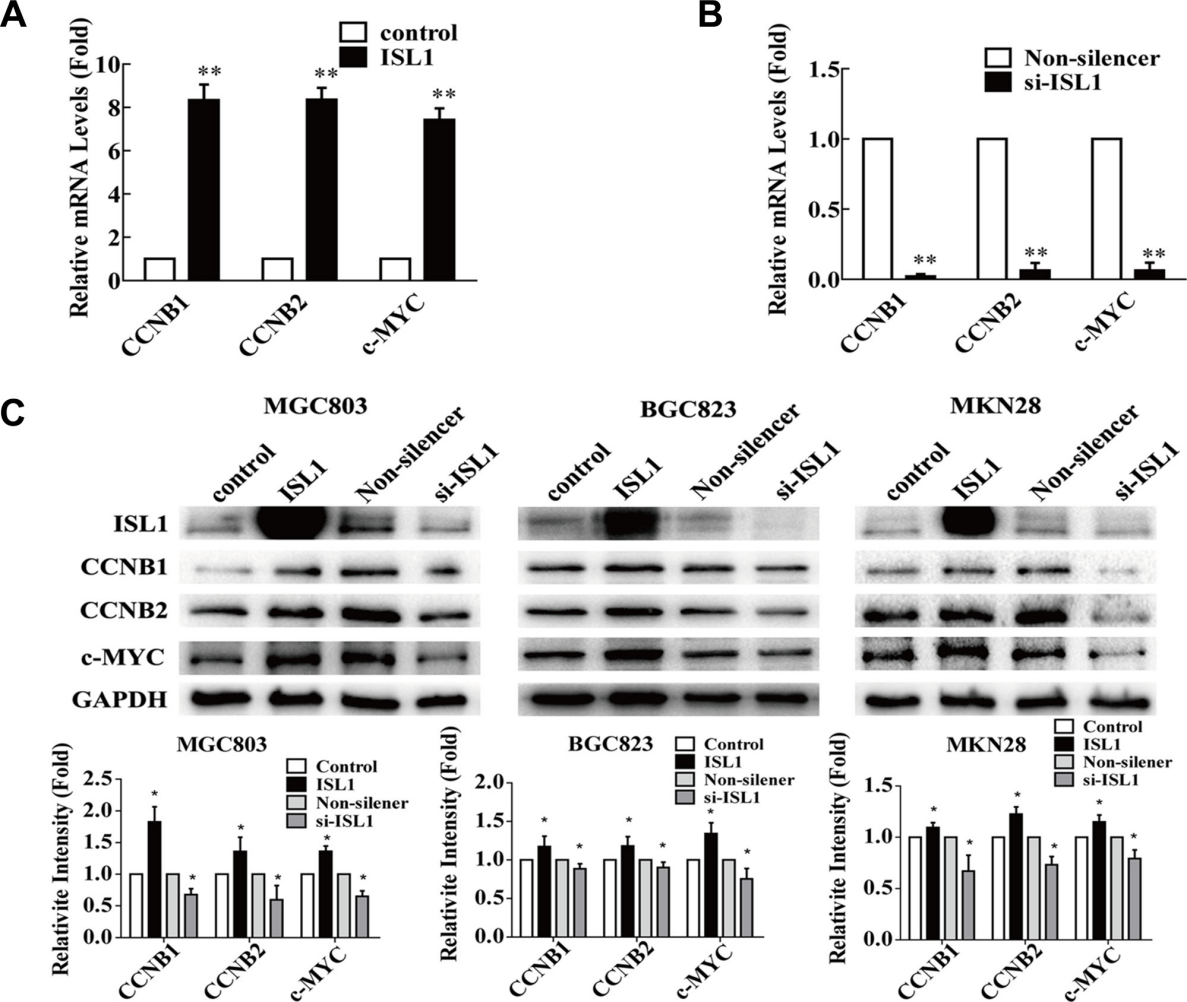
ISL1 regulated *CCNB1*, *CCNB2* and *c*-MYC expression (**A, B)** qRT-PCR analysis of *CCNB1*, *CCNB2* and *c-MYC* mRNA levels in MGC803 cells transfected with pcDNA3.1-ISL1 (ISL1) or pLL3.7-ISL1-siRNA (si-ISL1). The cells transfected with pcDNA3.1 (control) or pLL3.7-Non-silencer (Non-silencer, control) were used as the controls, whose values were set as 1. The data represent three independent experiments, each performed in triplicate. 18S rRNA levels served as the internal control. Bars represent the mean ± SD (*p < 0.05, **p < 0.01 vs. control). (**C)** Representative images of western blots of CCNB1, CCNB2 and c-MYC expression in the cells. The grayscale scanning was performed on the images from three independent experiments and the results are presented at the bottom. GAPDH levels served as the internal control. The values of the controls were set as 1. Bars represent the mean ± SD (*p < 0.05 vs. control or Non-silencer).

To confirm the relationship between ISL1 and the putative target genes, we analyzed the endogenous expression of *CCNB1*, *CCNB2* and *c-MYC* in *ISL1* overexpression or knockdown MGC803 cells (Supplementary Figure S2). The qRT-PCR results indicated that *ISL1* overexpression significantly increased the expression levels of the three genes by about 7–8-fold relative to the controls (*p* < 0.01 for *CCNB1* and *c-MYC*, *p* < 0.05 for *CCNB2*, Figure 6A); *ISL1* knockdown reduced their expression to less than 1/10 of the controls (*p* < 0.01, Figure 6B). Grayscale scanning analysis of the western blots from three independent experiments revealed that *ISL1* overexpression or knockdown had similar effects on the endogenous gene expression of *CCNB1*, *CCNB2* and *c-MYC* at protein levels in the MGC803, BGC823 and MKN28 cells (Figure 6C). Taken together, our findings show that *CCNB1*, *CCNB2* and *c-MYC* expression is positively correlated with *ISL1* expression.

## DISCUSSION

GC is the third leading cause of cancer death and remains a major clinical challenge due to its poor prognosis and limited treatment options [13]. Although intensive research has been conducted to detect and validate a variety of molecules associated with GC cell differentiation and proliferation [14], the molecular mechanisms are not fully understood.

In a previous study, we reported that high levels of *ISL1* expression may serve as a specific marker for GC treatment and prognosis [8]. However, how ISL1 functions in GC remains unknown. This prompted us to investigate the role of ISL1 in GC occurrence and development. In this study, we confirmed the aberrant expression of *ISL1* in GC tissues, which we had reported previously and its expression level is negatively correlated with the GC differentiation grade [8]. The GC cell lines had variable levels of ISL1 expression, where ISL1 expression was higher in MGC803 and BGC823 cells (both are of lower differentiation grade) and lower in MKN28 cells (a higher differentiation grade); ISL1 expression was lowest in the normal GES1 cell line, indicating that the differentiation grades of the cell lines were negatively correlated with the level of ISL1 expression. Moreover, the relationship between the ISL1 expression levels and proliferation rate in the GC cell lines was determined. The rates of cell proliferation were positively associated with *ISL1* expression levels; MGC803 cells expressed high level of *ISL1* and proliferated more quickly, and MKN28 cells expressed lower level of *ISL1* and proliferated more slowly; however, both GC cell lines proliferated more quickly than the GES1 cells. Furthermore, *ISL1* overexpression in MGC803 cells promoted xenograft growth *in vivo*, while *ISL1* knockdown inhibited tumor growth. We previously reported that ISL1 promoted NIH-3T3 cell tumorigenicity *in vivo*. In the present study, we examined whether ISL1 plays a role in GC tumor initiation and progression. However, *ISL1* overexpression in GES1 cells did not cause tumorigenesis following xenografting in BALB/c nude mice or in non-obese diabetic/severe combined immunodeficient (NOD/SCID) mice (data not shown), which does not support *ISL1* as an oncogene. Further investigations are required to elucidate the mechanism involved.

How does ISL1 promote GC proliferation and carcinogenesis? We found that ISL1 stimulated the cell cycle. Up- or downregulating *ISL1* in GC cells promoted cell cycle transition into the S/G_2_/M phases or promoted cell cycle arrest in the G_0_/G_1_ phases. More importantly, ISL1 can bind to *CCNB1*, *CCNB2* and *c-MYC* and consequently activates the cell cycle.

It has been established that c-MYC plays a key role in controlling cell proliferation [15]. Aberrant *c-MYC* expression is associated with poor survival in GC [16, 17]. In addition, CDK1–cyclin B complexes are critical for the proper temporal and spatial regulation of cellular proliferation [18, 19]. Cyclin B1 and cyclin B2 are the key factors to the initiation of mitosis, and can form complexes with CDK1 to regulate the G_2_/M phases [20]. Cyclin overexpression is linked to gastrointestinal cancers and is often associated with less favorable prognoses and outcomes [21]. High level of *CCNB1* expression has been detected in a variety of cancers [22–26], including GC [27], resulting in uncontrolled cell growth. A recent study reported that cyclin B1 and cyclin B2 were the most significant candidate biomarkers in GC [28].

In the present study, we identified *CCNB1*, *CCNB2* and *c-MYC* as the target genes of ISL1. It is well known that cyclin B1 is involved in G_2_-to-M transition and cyclin B2 is a G_2_/mitotic-specific factor. They both play a key role in the S-to-G_2_/M phases. The proto-oncogene *c-MYC* is a powerful promoter of cell cycle progression, including the G_1_/S phases. Thus, our results may explain why ISL1 promoted cell cycle transition either in G_0_/G_1_-to-S or in S-to-G_2_/M transition. Additionally, we noted that *c-MYC* was regulated more significantly by ISL1 than *CCNB1* and *CCNB2* were. This differs from the ISL1 regulation of its target genes in pancreatic islet cells we reported previously [10], where ISL1 regulated *CCND1* expression more significantly than it did on *c-MYC*. This implies that ISL1 may target different downstream genes in GC cells or normal islet cells and the reasons remain to be addressed.

In summary, we propose that ISL1 promotes tumor cell proliferation and tumorigenesis. ISL1 serves as a novel regulator for the expression of *CCNB*1, *CCNB*2 and *C-MYC*, which plays significant roles in GC progression and development. Therefore, our results suggest that ISL1 may serve as a potential biomarker for GC diagnosis and prognosis.

## MATERIALS AND METHODS

### Immunohistochemistry

All human GC tissue specimens were obtained from the Department of Pathology, School of Basic Medical Sciences, Peking University, with approval from the Peking University Research Ethics Committee (IRB 00001052–13014). The specimens were subjected to IHC analysis using an EnVision Detection Kit/DAB (GK500705, DAKO A/S, Glostrup, Denmark) according to the manufacturer's protocol with mouse monoclonal anti-ISL1 (ab86472; Abcam, Hong Kong, China). Monoclonal mouse immunoglobulin G (IgG)2a (X0943; DAKO A/S) was used as the isotope control.

### Cell lines and cell culture

GC cell lines BGC823 (ICLC: HTL98007), SNU-5 (ATCC: CRL-5971) and N87 (ATCC: CRL-5822) were purchased from Istituto Nazionale per la Ricerca sul Cancro (ICLC, Genova, Italy) and American Type Culture Collection (ATCC, Manassas, VA), respectively. MGC803, MKN28, MKN45 and an immortalized human gastric epithelial cell line, GES1, were gifts from Profs. Shou CC and Ke Y (Peking University Cancer Hospital). The cells were cultured in Dulbecco's modified Eagle's medium (DMEM) supplemented with 10% fetal bovine serum (FBS), 80 U/ml penicillin, and 100 mg/ml streptomycin.

### Cell transfection

To establish stable ISL1-overexpressing cell lines, 2 × 10^5^ cells were plated in a 60-mm culture dish. When approximately 50% confluent, the cells were transfected with 2 μg pcDNA3.1-ISL1 plasmid or control pcDNA3.1 plasmid with Lipofectamine 2000 (Invitrogen, Carlsbad, CA, USA). G418 selection (1000 μg/ml) was performed and single colonies were picked up at about 21 days after transfection. To establish stable ISL1 knockdown cell lines, the cells were transfected with pLL3.7-ISL1-siRNA (si-ISL1) or pLL3.7-Non-silencer as the control and then selected using puromycin resistance screening (0.5 μg/ml). Stable cell lines were characterized using quantitative reverse transcription-PCR (qRT-PCR) and western blotting to quantify the ISL1 expression levels.

### Cell proliferation assay and cell cycle analysis

Cell proliferation was measured using the CCK-8 kit (Dojindo Laboratories, Kumamoto, Japan) according to the manufacturer's instructions. For the CCK-8 assay, cells were seeded onto a 96-well plate at 1 × 10^3^ cells per well with 100 μl complete medium and cultured at 37°C; 10 μl CCK-8 solution was added to each well after 12 h, 24 h, 48 h, 72 h, and 96 h of culture, respectively. The plates were incubated at 37°C for 30 min, and then the absorbance at 450 nm was measured with a microplate reader (Bio-Rad, La Jolla, CA, USA). The values at each time point were normalized to that of the relative cells at time 0, which was set as 1. All experiments were performed in triplicate and three independent repeat experiments were performed. For cell cycle analysis, cells were fixed in ice-cold 70% ethanol and incubated at 4°C overnight. Fixed cells were washed with phosphate-buffered saline (PBS) and treated with 100 μg/ml RNase A at 37°C for 20 min. After staining with propidium iodide (50 μg/ml), the cells underwent fluorescence-activated cell sorting (FACS) on a FACScan instrument (Becton Dickinson, Franklin Lakes, NJ, USA). Cell debris and fixation artifacts were gated out and cell populations at the G_0_/G_1_, S, and G_2_/M phases were quantified using ModFit LT v2.0 software (Verity Software House, USA). All experiments were performed in triplicate and three independent repeat experiments were performed.

### Plate colony formation and soft agar colony formation assays

The plate colony formation (PCF) assay was carried out using 6-well plates. Cells (2 × 10^3^) were seeded in each well with 2 ml DMEM supplemented with 10% FBS. Culture medium was changed every three days. After 14 days, the resulting colonies were fixed with methanol at −20°C for 5 min, and then stained with crystal violet. Colonies > 4.5-mm diameter were counted and were analyzed using Image-Pro Plus (IPP 6.0) software. The colony formation index was defined as the ratio of colony numbers to initial numbers of the cells plated in each well (2000 cells/well). The index of the control cells was set as 1. Three independent experiments were performed, each in triplicate. For the soft agar colony formation (SACF) assay, cell suspensions were mixed with 0.3% soft agar in DMEM containing 10% FBS and layered in triplicate onto 0.6% solidified agar in DMEM containing 10% FBS (1 × 10^3^ cells/well). After 14 days of culture, the colonies in five fields per well were counted under a microscope.

### Xenograft tumor model

The animal experiments were performed in accordance with the ethical principles and guidelines for scientific experiments on animals of the Swiss Academy of Medical Sciences (1995). All protocols were approved by the Peking University Animal Care and Use Committee (LA 2010–066). Five-week-old BALB/c nude mice were purchased from the Peking University Department of Laboratory Animal Science. MGC803 cells (1 × 10^7^ cells suspended in 200 μl PBS) stably transfected with ISL1 or si-ISL1 plasmids (pCDNA3.1 or Non-silencer vector as the controls) were injected subcutaneously into the right oxter flank (*n* = 3 mice per group). All mice were maintained under specific pathogen–free conditions. Tumors were measured with calipers on the indicated days, and tumor volumes were calculated as 1/2 × length × width^2^. After the last measurement of tumor volume, the mice were sacrificed under anesthesia with 5 mg/100 g body weight sodium pentobarbital, and tumor tissues were removed. Whole-cell lysates of one tumor sample from each group were prepared and subjected to western blotting to detect ISL1 levels.

### qRT-PCR

Total RNA was isolated from cultured cells using an RNeasy mini kit (Qiagen, Hilden, Germany); complementary DNA (cDNA) was prepared from 1 μg total RNA with oligo (dT) primers using a SuperScript First-Strand cDNA Synthesis System (Invitrogen, Carlsbad, CA) according to the manufacturer's protocols. CDNA (10 ng) was used as the template for qRT-PCR using an Applied Biosystems 7500 Fast Sequence Detection System (Life Technologies Corporation, Carlsbad, CA). Relative mRNA expression was calculated from the comparative threshold cycle (ΔCt), and normalized to 18S rRNA. The primers used are listed in Table 1. All experiments were performed at least three times, each in triplicate.

**Table 1:** Primers used in qRT-PCR

Primers	Primer sequences (5′ - 3′)	Product size (bp)
*ISL1*	F: CTGCTTTTCAGCAACTGGTCA	128
	R: TAGGACTGGCTACCATGCTGT	
*c-MYC*	F: GCCACGTCTCCACACATCAG	141
	R: TCTTGGCAGCAGGATAGTCCTT	
*CCNB1*	F: TCCAGTTATGCAGCACCTGGCTA	162
	R: TGCCACAGCCTTGGCTAAATCTT	
*CCNB2*	F: TGGAAAAGTTGGCTCCAAAG	59
	R: CTTCCTTCATGGAGAGACATCCTC	
*18S rRNA*	F: GTAACCCGTT GAACCCCATT	151
	R: CCATCCAATCGGTAGTAGCG	

### Western blotting

Cell lysates were prepared using RIPA lysis buffer (P0013E; Beyotime, Shanghai, China) containing protease inhibitor cocktail (469313200; Roche, Basle, Switzerland). Western blot analysis was carried out as described previously [29]. Rabbit monoclonal anti-ISL1 antibody (ab109517) was purchased from Abcam. Mouse monoclonal anti–cyclin B1 (#4135), rabbit monoclonal anti–glyceraldehyde-3-phosphate dehydrogenase (GAPDH) (#2118), and anti–β-actin (#4970) were all purchased from Cell Signaling Technology. Mouse monoclonal anti-cyclin B2 (sc-28303) and rabbit polyclonal anti-c-myc (sc-764) were obtained from Santa Cruz Biotechnology. Grayscale scanning for western blots of three independent experiments was performed for quantitative analysis. β-Actin or GAPDH served as the internal controls.

### Luciferase assay

The pCDNA3.1-c-MYC, c-MYC-luc, and mutant-c-MYC-luc reporters were constructed previously by our laboratory [30]. The CCNB1-luc, CCNB2-luc, mutant-CCNB1-luc, and mutant-CCNB2-luc reporters were constructed by TransGen Biotech (Beijing, China). Plasmid transfection and luciferase activity detection were performed as described previously [30]. Briefly, MKN28 cells were seeded and cultured in a 24-well plate and transfected with the appropriate plasmid DNA using Lipofectamine 2000 (Invitrogen) according to the manufacturer's instructions when cells were 80% confluent. The total amount of DNA was kept constant using a pcDNA3 plasmid. After 48-h transfection, the cells were lysed and the luciferase activity was assayed. The luciferase activity was normalized to *Renilla* luciferase activity. Three independent repeat experiments were performed, each in triplicate.

### Chromatin immunoprecipitation

ChIP experiments were performed in MKN28 cells as previously described [31]. After elution, quantitative PCR (qPCR) was performed to amplify the DNA fragment containing the ISL1 binding sites on the *CCNB1* and *CCNB2* promoters and the *c-MYC* enhancer using the appropriate primers (Table 2).

**Table 2:** Primers used in ChIP assay

Primers	Primer sequences (5′ - 3′)	Product size (bp)
*CCNB1*	F: CCGCTTCGGACTGCGAACTAA	151
	R: AGAGCAGGCAGCAGCTAAGAAGG	
*CCNB2*	F: GCGGTATTTGAATCCTGGAACAAG	154
	R: CGGACTGAAAAGGGAGGACACT	
*c-MYC*	F: TACAGTGCACTTTCACTAGTATTCA	191
	R: TTATTGGAAATGCGGTCATG	

### Statistical analysis

Data are expressed as the mean ± standard deviation (SD). All statistical calculations were performed using SPSS17.0 for Windows (SPSS, Chicago, IL). The significance of differences between groups was determined using one-way analysis of variance (ANOVA). Differences were considered statistically significant at *p* < 0.05.

## SUPPLEMENTARY MATERIALS FIGURES


