# Supplementary material for: ISL1, a novel regulator of CCNB1, CCNB2 and c-MYC genes, promotes gastric cancer cell proliferation and tumor growth
Source: Oncotarget. 2016 May 10;7(24):36489–500. doi: 10.18632/oncotarget.9269 (PMC5095015; doi:10.18632/oncotarget.9269)
Supplement: Supplementary file 1 [file oncotarget-07-36489-s001.pdf]

ISL1, a novel regulator of *CCNB1*, *CCNB2* and *c-MYC* genes, promotes gastric cancer cell proliferation and tumor growth

Supplementary Materials

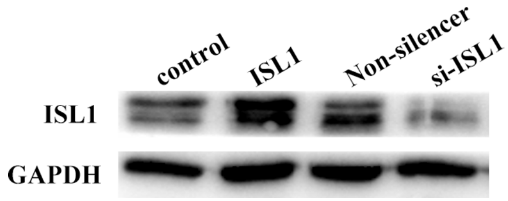

Supplementary Figure S1: Western blotting analysis of ISL1 protein levels in MGC803 cells stably transfected with pcDNA3.1-ISL1 (ISL1) or pLL3.7-ISL1-siRNA (si-ISL1) plasmids. Cells transfected with pcDNA3.1 (control) or pLL3.7-Non-silencer (Non-silencer) were used as the controls. GAPDH levels served as the internal control.

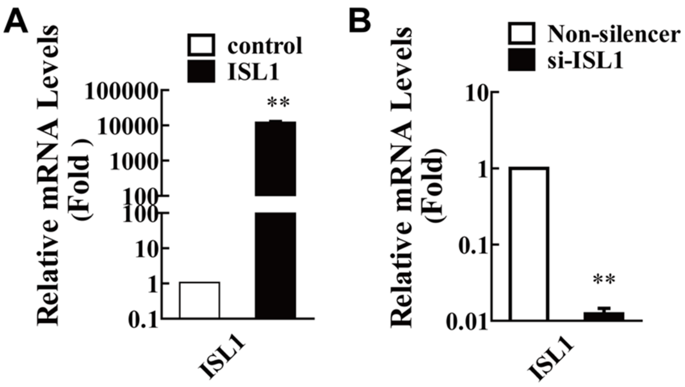

Supplementary Figure S2: (A, B) qRT-PCR analysis of *ISL1* levels in MGC803 cells transfected with pcDNA3.1-ISL1 (ISL1) or pLL3.7-ISL1-siRNA (si-ISL1). Cells transfected with pcDNA3.1 (control) or pLL3.7-Non-silencer (Non-silencer) were used as the controls. The data represent three independent experiments, each performed in triplicate. 18S rRNA levels served as the internal control. Bars represent the means ± SD (*$p < 0.05$, **$p < 0.01$ vs. control or Non-silencer).
